# Engage and enjoy–investigating predictors of employee engagement and work satisfaction in equine veterinary professionals

**DOI:** 10.3389/fvets.2023.1036388

**Published:** 2023-02-15

**Authors:** Yteke Elte, Kate Acton, Jessica Martin, Mirjam Nielen, René van Weeren, Inga Wolframm

**Affiliations:** ^1^Department of Clinical Sciences, Faculty of Veterinary Medicine, Utrecht University, Utrecht, Netherlands; ^2^The Royal (Dick) School of Veterinary Studies and the Roslin Institute, The University of Edinburgh, Edinburgh, United Kingdom; ^3^Department of Population Health Sciences, Faculty of Veterinary Medicine, Utrecht University, Utrecht, Netherlands

**Keywords:** veterinary practice, employee engagement, work satisfaction, human resource, equine practice, practice management, horse

## Abstract

**Introduction:**

Individuals working in the field of veterinary care are regularly affected by their profession. High levels of responsibility to often provide life-saving health care to animals combined with having to manage owners' expectations and irregular working hours can cause considerable levels of work-related stress among professionals in equine veterinary practice. On the positive side, research also shows that working in the veterinary profession can have a positive impact on personal wellbeing and feelings of fulfillment. A limited number of studies has investigated work satisfaction and engagement among veterinarians across the globe, and none specifically in the equine veterinary work field. The aim of the current study was to identify relevant predictors of employee engagement and work satisfaction in relation to demographic and work environment related factors in the equine veterinary profession.

**Methods:**

A cross-sectional study design was used to investigate work satisfaction and employee engagement among equine veterinary professionals from the UK, the US and the Netherlands using an online survey.

**Results:**

Results suggest that levels of work engagement and satisfaction in the veterinary profession may be gauged using four factors. These factors encompass Pride and purpose (the extent to which personal core values align with the mission of the employer, i.e., the veterinary practice), Company culture and relationship with management (the manner in which staff members interact with each other and the management), Working conditions and compensation (formal employment conditions relating to responsibilities and rewards and levels of collegiality) and Team culture and learning possibilities (encouragement to pursue personal and professional growth).

**Discussion:**

Findings underline the importance of being particularly mindful of inexperienced colleagues, those with demanding family commitments and, where feasible, of providing employees with a modicum of autonomy in order to ensure a satisfied equine veterinary workforce.

## Introduction

The veterinary profession has long been considered very stressful (1). High levels of responsibility to provide often life-saving health care to animals combined with having to manage the owners' expectations and irregular working hours can cause considerable levels of work-related stress (1). A relatively limited number of studies have investigated work satisfaction and engagement among veterinarians across the globe (2–4). These confirm that individuals working in the veterinary profession are regularly affected by their chosen profession. Most of the research has focused on adverse effects, such as stress, burn out and, in the worst case, suicide (1, 3, 5–12). However, studies by Cake et al. and Wallace et al., also examined the positive impact the veterinary profession can have on personal wellbeing and feelings of fulfillment (13–15).

While most of the available research on work satisfaction and engagement in the veterinary work force approaches the veterinary profession as a whole, little attention has been paid to the fact that the profession is highly diverse in terms of culture, legislation and practice. The limited assessment that has been undertaken has been primarily focused within the largest field of small animal practice (16). Veterinary communities are siloed based on professional focus such as small animal, equine, farm, exotic animals or outside of clinical practice (17). Differences in client characteristics and motivations for owning an animal lead to differences in animal-owner relationships, which are likely to cause the emergence of relevant subcultures within the different fields of veterinary practice (18). Furthermore, even within a particular sub-culture variations in clients' motivations to own an animal may lead to differences in how veterinarians approach and deal with their clients (18). In equine veterinary practice, veterinarians are required to cater to different types of clients, ranging from those who ride recreationally and consider their single horse as part of the family to those owning a stable of high-performance horses for competition at prestigious sporting events. The need to deal with differences in owner motivation and emotional attachment, as well as–at times conflicting–financial concerns put considerable pressure on veterinary professionals. They are required to adapt and evolve continuously in order to meet these various and varied demands of their clients (18). While some veterinarians might perceive such levels of professional dynamism as invigorating, others will experience difficulties in coping with them (19) and they may be further confounded by conflicting human-animal bond perspectives within the veterinary profession (20–22).

Previous work investigating depression, anxiety, stress and burnout in Australian veterinarians reported those working with equines as suffering the highest levels of depression (23). A decade later, equine veterinary organizations worldwide are currently voicing their concerns regarding the decrease in numbers of veterinary students choosing to go into equine veterinary practice and the high attrition rate of those working in the field (19). Therefore, the need to develop effective employment retention strategies has become a priority in the equine veterinary field (19). To be effective, these strategies should be informed by a thorough analysis of the current levels of work satisfaction and employee engagement within the sector.

The aim of the current study was to identify relevant predictors of employee engagement and work satisfaction in relation to demographic and work environment related factors in the equine veterinary profession. The outcome provides essential and informed input for the development of strategies to improve attractiveness and sustainability of the equine veterinary profession.

## Materials and methods

### Study design

A cross-sectional study design was used to investigate work satisfaction and employee engagement among equine veterinary professionals (veterinarians, veterinary nurses, practice management and support staff) using an online survey. The study protocol was approved by the Human Ethical Research Committee (HERC) at the Royal (Dick) School of Veterinary Studies, University of Edinburgh (HERC_250-18).

An online survey was hosted by Bristol Online Surveys (BOS) software (Jisc, Bristol, UK; https://www.jisc.ac.uk/#) and open from November 2018 till March 2019 to professionals working in equine veterinary practice. According to UK data protection laws and in compliance with GDPR, no personal or identifying information such as names, emails or IP addresses were collected. Participants were made aware before entering the survey that participation was voluntary and anonymous, and that they could withdraw from the study at any time when filling out the survey, however, after submission it would no longer be possible to withdraw (see Supplementary Table 1).

The online survey was distributed in the three principal target countries (The Netherlands, United States of America and United Kingdom) through newsletters of the Royal Dutch Veterinary Society (KNMvD), the American Association of Equine Practitioners (AAEP) and the British Equine Veterinary Association (BEVA). Further distribution was achieved *via* a snowball effect through sharing on relevant social media platforms (with permission) targeting all professionals working within (equine) veterinary practices (24). The survey was available in two languages: Dutch and English. Only participants that at the time of the study worked more than 50% in the equine work field were eligible for inclusion.

### Survey design

The survey consisted of two parts: (1) 16 questions on personal demographics and workplace characteristics; and (2) A detailed section including 31 work-related statements, which respondents were asked to rank their level of agreement for each (Supplementary Table 1). The section comprising work-related statements was based on previous research by Kumar et al. on work satisfaction and employee engagement (25, 26). Level of agreement was scored using a 5-point Likert scale. The survey was written in English and subsequently translated into Dutch by a native speaker and checked by a bilingual person. Respondents could choose to undertake the survey either in Dutch or English. Surveys in both languages were piloted to check for clarity of questions and online platform operation. The first page of the survey contained an introductory text to the survey and its purpose, followed by the informed consent statement which had to be actively ticked to indicate consent and provide access to the survey. The first question was about the respondent's proportion of work in equine practice. Those indicating <50% of working time focused on equines were immediately routed to the last page thanking them for their contribution. All others were routed through the remaining survey questions. Respondents who exited the survey prior to completion (e.g., exited the web browser) were removed prior to data analysis, ensuring that only completed responses were included. The survey took approximately 10 min to complete.

### Statistical analysis

Statistical analysis was performed using R software version 4.1.3 (R 47) through R Studio (2022.02.1 Build 461, RStudio, PBC, 2009–2022) (27). For statistical analysis, responses relating to identifying the purpose/relationship of clients' horses treated were re- categorized as either “sport” (combining original groups of “amateur sport,” “dressage,” “eventing,” “jumping” and “western”), “racing” (combining “harness” and “Thoroughbred racing”), and “leisure” (combining “leisure,” “companion” and “other”). Additionally, the respondents who were not resident within the target countries (i.e., United Kingdom, United States of America, Netherlands) were grouped into the “other” category.

For the 31 work-related statements, the 5-point Likert scale ranged from 1 = strongly disagree to 5 = strongly agree, with 3 being neutral. For data analysis, any answer at the central point (neither agree or disagree) was considered noninformative. For each question the original Likert scale minus the neutral midpoint was recoded 1 to 4 in order to prevent the emergence of a false central point that would inadvertently skew the data (28, 29). Multiple correspondence analysis (MCA) was conducted to assess the relationships between the levels of agreement for all statements, using packages FactoMineR and factoextra (30–32). The MCA allowed the underlying structures of the dataset to be assessed and factors (grouping of statements with similar profiles) to be identified based on eigenvalues, cumulative and proportion variances and pattern coefficients. This identified four factors, with a cumulative percentage (%) variance of 66.94%, with the first coordinate explaining the most variance (50.40%), the second coordinate explaining the next highest variance (7.14%), and so on. Cronbach's alpha was calculated to assess the reliability (i.e. internal consistency) of statement scale responses within a factor, using the “ltm” package (33), and demonstrated high intra-reliability (0.855–0.929) within each factor. Following MCA the factor profiles were labeled by the first and last author to reflect the collection of statements included per factor, increasing overall readability (Table 1). For the loading of all of the items on each of the four factors (see Supplementary Table 2).

**Table 1:** Multiple correspondence analysis (MCA) calculated factors, identifying the grouping of statements with similar agreement profiles, with factors labeled to encompass the collection of statements included.

**Work related factors**	**Pride and purpose**	**Company culture and relationship with management**	**Working conditions and compensation**	**Team culture and learning possibilities**
Description of content	Alignment of personal core values with the mission of the employer, i.e., the veterinary practice	The manner in which staff members interact with each other and the management	Formal employment conditions as they relate to responsibilities and rewards	Levels of collegiality and encouragement to pursue personal and professional growth
Cronbach's alpha per factor	0.884	0.929	0.856	0.855
	(0.869, 0.899)	(0.919, 0.937)	(0.837, 0.872)	(0.831, 0.876)
Individual survey items, (including standardized loadings)	*I am proud to tell people where I work. (0.83)*	*I feel like I have the all the support I need to meet my goals at work. (0.29)*	*My role here allows me to have an appropriate work life balance. (0.61)*	*Employees adapt quickly to difficult situations here. (0.68)*
	*The work we do here positively impacts our clients' lives. (0.97)*	*Senior management and employees trust and respect each other. (0.70)*	*I am satisfied with the amount of time and money the company invests in my CE (training courses offered by outside sources, courses, workshops, etc.) (0.30)*	*Employees here take the initiative to help other employees when the need arises. (0.78)*
	*I understand how my work impacts the company's business goals. (0.71)*	*Management recognizes strong job performance. (0.63)*	*I feel that I am compensated appropriately overall (including wage/salary and bonus, if applicable). (0.98)*	*Employees here are willing to take on new tasks as required. (0.85)*
	*When the company succeeds, I feel like the success is my own. (0.70)*	*Communication between management and employees is excellent here. (0.86)*	*I am satisfied with my total benefits package (wage, bonus, medical and dental plans, vacation days, paid personal/sick days–if applicable). (0.86)*	*I am satisfied with the internal (in-house) job-related training the company offers. (0.34)*
	*I understand how our Core Values (if applicable) relate to expectations around my behavior at work, and my work itself. (0.60)*	*I am happy with the overall culture of the company. (0.56)*	*I feel that the number and complexity of tasks and the general workload at the company are manageable*.	*Employees here willingly accept and embrace change. (0.59)*
	*I am content to spend the rest of my career with the company. (0.55)*	*I am involved in the decisions that affect my work here. (0.88)*	*I am compensated fairly relative to similar/the same positions in similar businesses in my area. (0.79)*	*My co-workers and I have an excellent working relationship. (0.76)*
	*I get excited about going to work. (0.58)*	*I have a sense of ownership and pride in the company. (0.58)*	*I am confident that the method used to determine my wage or salary increases on a yearly basis is fair. (0.55)*	
	*I am satisfied with my opportunities for growth within the company. (0.44)*	*I feel like the management and staff of the company adhere to the Core Values (if applicable). (0.51)*	*I am satisfied by the workplace flexibility offered by the company. (0.31)*	

Cronbach's alpha [inc. 95% confidence intervals (CI)] demonstrate the intra-reliability of statement scale responses within a factor.

Linear models (lme4) were used to detect aspects (e.g., demographics, horses treated, etc.) which might affect the identified four factors representing work satisfaction attributes (34). Model fit was determined by examination of residuals *via* the DHARMa package (35). All demographic and practice parameters were included as fixed effects in maximal models. We assessed significance of explanatory variables with the ANOVA function in the car package, with statistical significance set at *p* < 0.05 (36). Following maximal model approaches, all variables were offered to the models, and all possible interactions between primary fixed effects were included in the models. According to step-wise regression reduction, insignificant non-primary research fixed factor interactions were removed (37). Variables were excluded based on automated backward selection process. Pairwise comparisons were conducted using the emmeans package and reported as estimated marginal means with *P* values adjusted for multiple comparisons using the Tukey method (38).

## Results

### General aspects of response and demographic profile of respondents

A total of 518 complete responses to the survey were recorded, providing informed consent, and meeting the criteria of predominantly working with equines. Most responses were from the primarily targeted countries [United Kingdom (UK), 37%, *n* = 189; the Netherlands (NL), 11%, *n* = 58 and the United States (US), 35%, *n* = 183]. An additional 88 responses (17%) were recorded from in total 29 non-target countries (see Supplementary Table 3 for exhaustive list of countries with corresponding number of respondents). Most respondents were female (84%, *n* = 437), varying between 74 and 90% between the three target countries (UK 85%, NL 74% and US 90%). Overall, most respondents were aged between 18 and 39 years of age (69%, *n* = 358). In the 5-level age classification (18–29; 30–39; 40–49; 50–59; and 60+ years old), across all target countries the largest number of responses (~40%) came from the 30-39 years old group (UK 42%; NL 38%; US 42%). For UK (37%, *n* = 70) and NL (22%, *n* = 13), the second largest age group was the youngest (18–29 years old), while for the US it was the 40–49 years old (22%, *n* = 41). Across all countries, equine professionals aged over 60 years old represented the smallest number in the survey (<4%). Most respondents reported their relationship status as “in a relationship” (72%, *n* = 374), with the remaining reporting as “single” (27%, *n* = 140), or provided no response (1%, *n* = 4). The number of respondents reporting to have dependents (i.e., children) was 29% (*n* = 148) overall. Similar percentages were seen in the UK (25%, *n* = 47) and the US (26%, *n* = 48), however in the Netherlands the percentage of equine professionals with dependents was markedly higher (48%, *n* = 28). Of the respondents with dependents, over 80%, irrespective of country, reported as being currently in a relationship, with the UK (96%) and the Netherlands (93%) showing the highest proportion of respondents with children also currently in a relationship.

### Working environment

Most respondents reported their work status as employee (81%, *n* = 420), 19% classified as employer (*n* = 98). Employee representation was highest in the UK (93%, *n* = 175) compared to 76% (*n* = 44) in the Netherlands and 72% (*n* = 131) in the USA. Most respondents, irrespective of country, reported working in general practice/ambulatory care (64%, *n* = 329) with the remaining working in hospitals or referral centers (24%, *n* = 126). Most respondents worked only with horses (77%, *n* = 398), the remaining in mixed practice. Irrespective of this, ~90% of respondents reported working in all three sectors (racing (90.0%), leisure (89.2%) and sport (88.8%). Country differences were marginal, with racing horses being the primary patient (91%) in the US, whilst in the Netherlands and the UK (both 95%) leisure horses were the primary patients.

Sixty-four percent (*n* = 329) qualified as veterinarians (UK 77%, NL 64%, US 49%). The proportion of respondents reporting as either registered veterinary nurses (RVNs) or technicians was consistent across all three countries (8%). The other respondents identified as either non-veterinarian managers, practice owners, administrative staff or other. The proportion of this category was relatively high in the US (43%, against <30% in UK and NL).

Respondents from the Netherlands and the US were grossly evenly divided over the two classifications of experience (<5 or 5+ years), irrespective of role, while the majority from the UK (67%, *n* = 126) had <5 years' experience. Importantly, when focusing only on veterinarians, approximately 70% of the respondents from the UK and US reported their experience as being <5 years. In the Netherlands this proportion was smaller (59%, *n* = 21).

### Factors determining employee engagement and work satisfaction

#### Work satisfaction attribute: Pride and purpose

Overall work satisfaction (higher agreement with statements) according to the factor *pride and purpose* was significantly higher for employers compared to employees (*p* < 0.0001; see Table 2). Respondents identifying as veterinarians showed lower work satisfaction within *pride and purpose* compared to non-veterinarians. Fixed effects representing respondents working with race or leisure horses were dropped during stepwise model reductions, however, working with sport horses (amateur and dressage) was shown to be associated with higher work satisfaction according to *pride and purpose* compared to those who did not. There was no effect of country or age on work satisfaction related to the *pride and purpose* factor. Finally, the interaction between relationship status and having dependents had an effect on *pride and purpose* work satisfaction. For those without children, work satisfaction was higher for those who were single compared to those who were in a relationship.

**Table 2:** Effect of factor “pride and purpose” on overall work satisfaction for job status, veterinarian role, horse category and relationship status (with/without dependents).

	**Estimate**	**Mean**	**SE**	**df**	**Lower CL**	**Upper CL**	**t ratio**	***p* value**
**Job status**								
Employee	n.a.	−0.211	0.104	502	−0.415	−0.00621	n.a.	n.a.
Employer	n.a.	0.518	0.136	502	0.25	0.78501	n.a.	n.a.
Difference employee vs. employer	−0.782	n.a.	0.137	502	n.a.	n.a.	−5.303	<0.0001
**Veterinarian role**								
Non vet	n.a.	0.2802	0.107	502	0.0697	0.491	n.a.	n.a.
Veterinarian	n.a.	0.0269	0.119	502	−0.2077	0.261	n.a.	n.a.
Difference non vet vs. veterinarian^*^	0.253	n.a.	0.108	502	n.a.	n.a.	2.349	0.0192
**Horse category**								
Non sport	n.a.	0.0184	0.1417	502	−0.271	0.308	n.a.	n.a.
Sport horses	n.a.	0.2886	0.0832	502	0.125	0.452	n.a.	n.a.
Difference non sport vs. sport horses^*^	−0.27	n.a.	0.132	502	n.a.	n.a.	−2.043	0.0416
**Relationship status (no dependents)**								
Relationship	n.a.	0.105	0.0922	502	−0.0767	0.286	n.a.	n.a.
Single	n.a.	0.317	0.1108	502	0.0996	0.535	n.a.	n.a.
Difference relationship vs. single^*^	−0.213	n.a.	0.103	502	n.a.	n.a.	−2.058	0.0401
**Relationship status (with dependents)**								
Relationship	n.a.	0.312	0.1002	502	0.1157	0.509	n.a.	n.a.
Single	n.a.	−0.12	0.284	502	−0.6783	0.438	n.a.	n.a.
Difference relationship vs single	0.433	n.a.	0.286	502	n.a.	n.a.	1.511	0.1315

Confidence level used: 0.95; ^*^p < 0.05.

#### Work satisfaction attribute: Company culture and relationship with management

Overall work satisfaction (higher agreement with statements) according to *company culture and relationship management* was significantly higher for employers compared to employees (*p* < 0.0001; see Table 3). Respondents identifying as veterinarians showed lower work satisfaction within the *company culture and relationship management* factor compared to non-veterinarians. Those who work with sport horses (amateur and dressage) were shown to be associated with higher work satisfaction according to *company culture and relationship management* compared to those who did not. There was no significant effect of country, age or relationship status on work satisfaction related to the *company culture and relationship management* factor.

**Table 3:** Effect of factor “company culture and relationship with management” on overall work satisfaction for job status, veterinarian role, horse category and relationship status (with/without dependents).

	**Estimate**	**Mean**	**SE**	**df**	**Lower CL**	**Upper CL**	**t ratio**	***p* value**
**Job status**								
Employee	n.a.	−0.402	0.0992	504	−0.597	−0.207	n.a.	n.a.
Employer	n.a.	0.626	0.1276	504	0.376	0.877	n.a.	n.a.
Difference employee vs. employer	−1.03	n.a.	0.129	504	n.a.	n.a.	−7.969	<0.0001
**Veterinarian role**								
Non vet	n.a.	0.214	0.1	504	0.0173	0.411	n.a.	n.a.
Veterinarian	n.a.	0.0104	0.133	504	−0.2123	0.233	n.a.	n.a.
Difference non vet vs. veterinarian^*^	0.204	n.a.	0.101	504	n.a.	n.a.	2.02	0.0439
**Horse category**								
Non sport	n.a.	0.0523	0.1367	504	−0.3209	0.216	n.a.	n.a.
Sport horses	n.a.	0.2768	0.0935	504	0.0932	0.46	n.a.	n.a.
Difference non sport vs. sport horses^*^	−0.329	n.a.	0.139	504	n.a.	n.a.	−2.372	0.0181
**Relationship status (no dependents)**								
Relationship	n.a.	0.0767	0.087	504	−0.0942	0.248	n.a.	n.a.
Single	n.a.	0.2756	0.1054	504	0.0686	0.483	n.a.	n.a.
Difference relationship vs. single	−0.131	n.a.	0.0983	504	n.a.	n.a.	−1.329	0.1844
**Relationship status (with dependents)**								
Relationship	n.a.	0.2073	0.0996	504	0.0117	0.403	n.a.	n.a.
Single	n.a.	−0.1106	0.2662	504	−0.6337	0.412	n.a.	n.a.
Difference relationship vs single	0.386	n.a.	0.2775	504	n.a.	n.a.	1.392	0.1645

Confidence level used: 0.95; ^*^p < 0.05.

#### Work satisfaction attribute: Working conditions and compensation

Overall work satisfaction (higher agreement with statements) according to *working conditions and compensation* was significantly higher for employers compared to employees (*p* < 0.0001; see Table 4). Older respondents (age range 40–60+ years) showed higher work satisfaction within the *working conditions and compensation* factor compared to younger respondents (age range 18–39 years). There was no significant effect of country, working with sport horses, relationship status or job title on work satisfaction related to the *working conditions and compensation* factor.

**Table 4:** Effect of factor “working conditions and compensation” on overall work satisfaction for job status, experience, age and horse category.

	**Estimate**	**Mean**	**SE**	**df**	**Lower CL**	**Upper CL**	**t ratio**	***p* value**
**Job status**								
Employee	n.a.	−0.0471	0.0893	503	−0.222	0.128	n.a.	n.a.
Employer	n.a.	0.4507	0.1277	503	0.2	0.701	n.a.	n.a.
Difference employee vs. employer	−0.498	n.a.	0.126	503	n.a.	n.a.	−3.938	0.0001
**Experience**								
<5 years	n.a.	0.296	0.11	503	0.079	0.512	n.a.	n.a.
5+ years	n.a.	0.108	0.096	503	−0.8807	0.296	n.a.	n.a.
Difference experience (<5 years - 5+ years)	0.188	n.a.	0.101	503	n.a.	n.a.	1.861	0.0634
**Age**								
18–39 years	n.a.	0.0319	0.102	503	−0.168	0.232	n.a.	n.a.
40–60+ years	n.a.	0.3717	0.11	503	0.155	0.589	n.a.	n.a.
Difference age (18–39 years)-(40–60+ years)^**^	−0.34	n.a.	0.112	503	n.a.	n.a.	−3.031	0.0026
**Horse category**								
Non sport	n.a.	0.0835	0.1433	503	−0.198	0.365	n.a.	n.a.
Sport horses	n.a.	0.3201	0.0714	503	0.18	0.46	n.a.	n.a.
Difference non sport vs. sport horses	−0.237	n.a.	0.137	503	n.a.	n.a.	−1.729	0.0845

Confidence level used: 0.95; ^**^p < 0.01.

#### Work satisfaction attribute: Team culture and learning possibilities

Overall work satisfaction (higher agreement with statements) according *to team culture and learning possibilities* was significantly higher for employers compared to employees (*p* = 0.0061; see Table 5). Overall country differences showed the UK having the highest satisfaction, and pairwise differences identified satisfaction was significantly higher for those from the UK compared to those from the USA according to *team culture and learning possibilities* factor. Those with less job experience (<5 years) showed higher work satisfaction within the *team culture and learning possibilities* factor compared to those who had worked longer than 5 years.

**Table 5:** Effect of factor “team culture and learning possibilities” on overall work satisfaction for job status, experience, horse category and relationship status (with/without dependents).

	**Estimate**	**Mean**	**SE**	**df**	**Lower CL**	**Upper CL**	**t ratio**	***p* value**
**Job status**								
Employee	n.a.	−0.238	0.109	501	−0.454	−0.0233	n.a.	n.a.
Employer	n.a.	0.162	0.145	501	0.122	0.447	n.a.	n.a.
Difference employee vs. employer^**^	−0.401	n.a.	0.145	501	n.a.	n.a.	−2.756	0.0061
**Experience**								
<5 years	n.a.	0.0702	0.124	501	0.174	0.3144	n.a.	n.a.
5+ years	n.a.	0.1463	0.112	501	−0.366	0.0736	n.a.	n.a.
Difference experience (<5 years - 5+ years)^*^	0.216	n.a.	0.106	501	n.a.	n.a.	2.05	0.0409
**Horse category**								
Non sport	n.a.	−0.228	0.156	501	−0.5342	0.0786	n.a.	n.a.
Sport horses	n.a.	0.152	0.0881	501	−0.214	0.3249	n.a.	n.a.
Difference non sport vs. sport horses^**^	−0.38	n.a.	0.139	501	n.a.	n.a.	−2.726	0.0066
**Relationship status (no dependents)**								
Relationship	n.a.	−0.0894	0.097	501	−0.28	0.101	n.a.	n.a.
Single	n.a.	0.114	0.117	501	−0.115	0.343	n.a.	n.a.
Difference relationship vs. single	−0.2	n.a.	0.114	501	n.a.	n.a.	−1.75	0.0807
**Relationship status (with dependents)**								
Relationship	n.a.	0.1106	0.109	501	0.103	0.324	n.a.	n.a.
Single	n.a.	−0.2874	0.3	501	−0.877	0.302	n.a.	n.a.
Difference relationship vs. single	0.401	n.a.	0.309	501	n.a.	n.a.	1.297	0.1952

Confidence level used: 0.95; ^*^p < 0.05; ^**^p < 0.01.

Those who work with sport horses (amateur and dressage) were shown to be associated with higher work satisfaction (though the trend is borderline/non-significant) according to *team culture and learning possibilities* compared to those who did not (leisure and racing). There was no effect of relationship status, age or job title on work satisfaction related to the *team culture and learning possibilities* factor.

## Discussion

The aim of the current study was to identify relevant predictors of employee engagement and work satisfaction within the equine veterinary profession while assessing the contribution of personal demographics and workplace characteristics.

Results from the current study suggest that levels of work engagement and satisfaction (for the purpose of brevity referred to as “work satisfaction” from here on) in the veterinary profession may be gauged using four factors. These factors encompass the extent to which personal core values align with the mission of the employer, i.e., the veterinary practice (*pride and purpose*), the manner in which staff members interact with each other and the management (*company culture and relationship with management*), formal employment conditions as they relate to responsibilities and rewards (*working conditions and compensation*) and levels of collegiality and encouragement to pursue personal and professional growth (*team culture and learning possibilities*). Significant predictive interactions of these factors with relevant personal and professional demographics provide key insights into the dynamics of equine veterinary professionals.

In all four factors “employment status” was predictive of overall work satisfaction, with employers demonstrating higher levels than employees, indicating that the ability to influence or control work related matters is highly important to equine veterinary professionals. In their seminal work on Self Determination Theory (SDT), Deci and Ryan proposed that in addition to relatedness and competence, autonomy, i.e., the ability to control one's own actions and behaviors, is one of the three psychological needs essential for effective human functioning and wellness (39, 40). In this same line, in her study on the role of personal resources in explaining wellbeing and performance among young veterinary professionals Mastenbroek found autonomy to be one of the central tenets with regard to the deployment of both job and personal resources (41).

Employees are likely to be less self-directing and will have to comply with the demands set by management. Depending on whether these demands align with an employee's own aspiration and motivation, these demands may cause an employee a considerable level of stress. Employers, on the other hand, may experience greater levels of autonomy, as they can determine the direction of the company, their personal focus as well as how they choose to engage with the people they lead (42, 43). It could be argued, therefore, that the feelings of innate satisfaction derived from the level of autonomy experienced offset any negative emotional responses that might be associated with running a business.

A growing body of evidence has shown the impact of leadership on work performances and satisfaction through its influence on human motivation (43). Different behaviors of leaders and leadership styles, such as transformational leadership, charismatic leadership, authentic leadership and ethical leadership are associated with different effects on engagement (44). It requires more specific investigations to determine which leadership styles have the most positive effect on employee engagement in equine veterinary practice.

Work satisfaction in terms of *Team culture and Learning possibilities* was higher in participants from the UK compared to the other countries. Research by Cake et al. demonstrated that, when setting out in their career, veterinary graduates are motivated not only by affiliations with animals, but are also attracted by the prospect of intellectual challenges and being able to interact with others (45). It stands to reason, therefore, that whenever veterinary practices actively promote professional development opportunities and foster an atmosphere of social support, they are being rewarded with veterinary professionals who are more satisfied. In the UK, veterinary practices have been actively encouraging veterinary staff programs, focusing on the development of both technical and “soft” skills (45). Additional research should determine whether these types of programs might be more effective than professional development programs in other countries in terms of enhancing levels of work satisfaction.

Work satisfaction according to *Company culture and relationship with management* and *Pride and purpose* was shown to depend on the type of profession with veterinarians experiencing significantly lower work satisfaction than non-veterinarians. In a German study veterinarians were found to have lower work satisfaction compared to general population subgroups with similar professional positions and educational levels, reportedly caused by dissatisfaction with long working hours (especially in equine work) and low income (2). Work satisfaction has long been considered an important predictor of performance. Improving work satisfaction is therefore likely to enhance a practice's organizational effectiveness and discourage withdrawal behaviors such as employee turnover and absenteeism (5, 6).

In individuals without a partner (and without dependents) work satisfaction according to Pride and Purpose was highest. Prior research demonstrated that responsibilities regarding family and housework can conflict with work roles, which can negatively impact work satisfaction (5). Highest levels of job strain were found in married females (46). Previous work on factors influencing work-life balance found that single employees with no dependents reported a better work-life balance than couples (with or without children) and single parents (14, 46, 47). The same study showed that a higher rated work-life balance correlated positively with overall employee engagement. Feelings of relatedness toward work colleagues are thought to positively affect work motivation, which, in turn, facilitates work satisfaction but prevents emotional exhaustion (48). It can be conjectured that, in the absence of a partner at home, interpersonal relationships in the workplace become increasingly important. The need to feel connected to others, i.e., to experience “relatedness,” is essential to human wellbeing and will express itself in the setting most readily available (40). It is important to note that the current study did not define the term “dependent.” Respondents might have been referring to adult dependents as well as children. Additional research will need to study the effect of different types of dependents on work engagement.

With regard to work experience, levels of work satisfaction according to *Team culture and Learning possibilities* were highest in participants with <5 years' experience. To them, support of their colleagues significantly increased work satisfaction, making them greet new challenges with enthusiasm. Other studies have reported the early phase in a career to be particularly stressful, leading to high levels of anxiety in inexperienced professionals (23, 49, 50). During the first five years, young veterinary surgeons are unsure of their skills and how to apply theoretical knowledge into practice. Therefore, it stands to reason that an atmosphere of collegiate support and a “can do” mentality will help ease young veterinary professionals into their careers. All the more important therefore, to not only prevent veterinarians from leaving the profession, but also to develop strategies to encourage more veterinary students to pursue an equine related career.

Work satisfaction in terms of *working conditions and compensation* also depended on levels of experience. This time though, more experienced colleagues scored higher, mirroring earlier research investigating the influence of age on job satisfaction (51). Older employees might have developed better coping strategies against work related stress, while those unable to cope might already have given up their work as a veterinarian (8). Another explanation might be found in generational differences, as younger generations can have different attitudes toward work than earlier generations. Sixty-nine percent of the respondents in the current study are under the age of 40. Those born between 1981 and 2000 are known as millennials or Generation Y (52). Studies report that factors such as flexibility in working hours (as opposed to fewer working hours), having meaningful work and being able to maintain an appropriate work-life balance rate particularly highly among the millennial generation (52). Even though additional research is required, employers and managers should acknowledge the existence of generational differences and take these into account to optimize job satisfaction for those they lead.

Helping animals and people are two job characteristics that contribute to a sense of meaning for veterinarians (15). Current findings suggest that the type of horse treated may be an additional factor. Veterinarians working with sport horses experienced significantly higher levels of work satisfaction than those focusing on racehorses. Seeing that sport and leisure horses are generally kept for longer and out of feelings of love or friendship, it could be argued that providing veterinary care to these horses, rather than race horses, aligns more closely with a veterinarian's intrinsic motivation (53). While preliminary work suggests that jockeys develop emotional attachments with their rides, the horses' owners are responsible for the horse's veterinary care (54). It might be argued that racehorse owners approach veterinary care more from a cost-benefit perspective, making it less appealing to veterinarians (55). However, additional research is required to explore such notions further.

## Limitations

As is the case with most, if not all questionnaires, the current study might have been prone to self-assessment bias. Participants may also have been drawn to participate in the study due to pre-existing levels of dissatisfaction in their work (56). On the other hand, completing a survey takes time and the mental energy to engage with the topic at hand. Veterinary professionals with little time and greater levels of dissatisfaction might not have felt the inclination to participate. Using a Likert scale can also lead to information bias as respondents may give socially desirable or acquiescent answers (53, 54).

In the analysis, the Likert scales were recoded into 1–4 after eliminating the neutral answers. This was done per question, which limited the loss of valid answers. Nevertheless, we suggest that in future studies on work related opinions researchers utilize Likert scales with even options, thereby preventing participants to provide noninformative answers (29). The survey only provides the options “employee” or “employer,” which, strictly speaking does not account for self-employed individuals or solo practitioners. Presumably they categorized themselves as “employer,” yet any generalization of results should be done with care. What is more, the study design did not account for the views of veterinarians who may have left the industry prematurely. Their viewpoint might have shed additional light on the situation.

Due to new EU privacy regulations, it was not possible to target equine veterinary professionals directly *via* email. The questionnaire therefore had to be distributed through digital newsletters from relevant organizations and social media channels, making response rates rather unpredictable. What is more, willingness to participate in online research declines with age, with people over 45 years of age being more inclined to participate in in-person surveys (57). This could also have led to an overrepresentation of younger participants, with fewer than 5 years' experience, making the results less predictable for the whole population of individuals working in equine veterinary practice.

Lastly, 84.4% of the respondents to the survey identified as female. While such findings might be evidence of the feminization of the veterinary population, they may also be indicative of a more general trend of online surveys being completed significantly more by women (58). While the statistical analysis demonstrated no gender effects, this may be due to the large female bias in the response. To prevent any misinterpretation, no subsequent comparison between male or female was made. Any future research should focus on recruiting a more balanced representation of male and female veterinary practitioners.

## Conclusion

Equine veterinary organizations worldwide are voicing their concerns regarding, on the one hand, the decreasing number of veterinary students choosing to go into equine veterinary practice and, on the other, the high attrition rate of those working in the field. Reportedly high stress levels, burn out and subsequent staff shortages, combined with ever demanding clients all contribute to a sense of urgency regarding the sustainability of the (equine) veterinary work field. With human capital being the most important asset–in the veterinary industry as much as in any other fields of work–the current research has aimed to contribute to the body of knowledge as it relates to work satisfaction and employee engagement in equine veterinary professionals worldwide.

Current findings suggest that levels of work satisfaction in the equine veterinary profession may be gauged using four factors. These factors encompass the extent to which personal core values align with the mission of the employer, i.e., the veterinary practice (*pride and purpose*), the manner in which staff members interact with each other and the management (*company culture and relationship with management*), formal employment conditions relating to responsibilities and rewards (*working conditions and compensation*) and levels of collegiality and encouragement to pursue personal and professional growth (*team culture and learning possibilities*).

While the outcomes of this study are undoubtedly complex, there are a number of starting points that may be used when designing relevant strategies to encourage greater work satisfaction, and, ideally, levels of employee retention. Current findings underline the importance of being particularly mindful of inexperienced colleagues, those with demanding family commitments and, where possible and feasible, of providing employees with a modicum of autonomy. As it stands, and perhaps not altogether surprisingly, veterinary employers experience greater work satisfaction than employees. However, not everyone can be the boss. To make sure that work satisfaction remains high, veterinary professionals at the start of their career should be made to feel part of a team and be given ample opportunity to develop additional skills. More seasoned colleagues, on the other hand, should be compensated appropriately, with working conditions that fit in with their lifestyle as they considered levels of compensation and appealing working conditions to be more important. Equine veterinary professionals who find themselves without a partner or children should make sure that their personal values align closely with the core mission of the veterinary practice they work for. Lastly, the type of client, or rather type of horse, may also play a role: sport horses, or so it seems, are generally preferred over racehorses.

To conclude, while it is impossible for an employer to control for every variable, knowing how personnel demographics and work characteristics might affect perceptions of work engagement can go a long way toward creating a better working environment. Most importantly perhaps, the issue of veterinary retention is not isolated to any particular country. Therefore, a coordinated multi-national, multi-stakeholder approach might go a considerable way toward a more satisfied equine veterinary workforce worldwide.

## Data availability statement

The datasets presented in this study can be found in online repositories. The names of the repository/repositories and accession number(s) can be found below: http://rpubs.com/jess_hop_bird/988771.

## Ethics statement

The studies involving human participants were reviewed and approved by Human Ethical Research Committee (HERC) at the Royal (Dick) School of Veterinary Studies, University of Edinburgh (HERC_250-18). The patients/participants provided their written informed consent to participate in this study.

## Author contributions

All authors listed have made a substantial, direct, and intellectual contribution to the work and approved it for publication.
